# Correlation between Fitness and Fatness in 6-14-year Old Serbian School Children

**DOI:** 10.3329/jhpn.v29i1.7566

**Published:** 2011-02

**Authors:** Sergej M. Ostojic, Marko D. Stojanovic, Vladan Stojanovic, Jelena Maric, Nenad Njaradi

**Affiliations:** ^1^ Biomedical Sciences Department, Faculty of Sport and Tourism, Novi Sad, Metropolitan University, Belgrade, Serbia; ^2^ Exercise Physiology Laborato**r**y, City Center for Physical Culture, Belgrade, Serbia

**Keywords:** Body-fat, Cross-sectional studies, Fitness, Girls, Obesity, Serbia

## Abstract

Lack of physical activity and/or physical fitness are some reasons epidemiologists suggest for increase in childhood obesity in the last 20 years, with clear correlation between body composition and physical activity and/or physical fitness yet to be determined. The objectives of the study were to (a) investigate the prevalence of overweight and obesity among Serbian school children and (b) determine the relationship between indicators of physical activity and body fatness in Serbian school children aged 6-14 years. The study subjects included a representative sample of Serbian elementary school children (n=1, 121—754 boys and 367 girls—aged 6.2-14.1 years), all of whom were recruited in the OLIMP (Obesity and Physical Activity among Serbian School Children) study. Anthropometric and physical fitness values, including body mass index (BMI), waist-circumference, body-fat, and aerobic capacity, were measured in all the children. Significant differences were found between male and female children regarding the prevalence of obesity (6.8% vs 8.2%, p<0.05, boys and girls respectively). Boys had significantly lower body mass, BMI, waist-circumference, sum of six skinfolds, and body-fat compared to their female counterparts (p<0.05). The highest level of weight, BMI, body-fat, and waist-circumference observed in a 14-year old girl (96.3 kg, 40.5 kg/m^2^, 54.5%, 91.4 cm respectively) implies the existence of extreme obesity in Serbian school children. The negative relationship between body-fat and maximal oxygen (VO_2_max) uptake was moderately high (r=-0.76; p<0.05). The study has shown a high prevalence of adiposity among Serbian school children, with a strong negative relationship between aerobic fitness and body fatness. Data of the study emphasize the necessity to identify children with weight problems and to develop early interventions to improve physical activity in children and prevent the increase of childhood obesity.

## INTRODUCTION

The prevalence of childhood obesity has been increasing dramatically worldwide, particularly in the last two decades (1–3). Although the prevalence of overweight and obesity varies quite substantially across ethnic groups and gender, numerous studies have shown alarmingly high levels of being fat among children. Estimates in several studies indicate that one in four children aged 6-14 years is presently overweight in developed and developing countries (4, 5), which ranges from 11% to 39% (6–9). The prevalence of overweight and obesity was found similar (9) or significantly different (10) between genders, with difference found in studies that reported higher prevalence of overweight and obesity in either girls (11–13) or boys (14). It is unclear what cultural, lifestyle, genetic, or environmental factors may explain these differences (15). The level of prevalence varies substantially with geographical region in European school children and is reported to be as high as 27.7% and 28% for boys and girls of the Eastern region respectively (16). To the best of our knowledge, to date, no published data are available regarding the prevalence rates of overweight and obesity for Serbian school children.

Lack of physical activity and/or physical fitness and excessive calorie consumption are some reasons epidemiologists suggest for the increase of obesity in the last 20 years (4, 17). The low level of physical activity and health-related physical fitness, represented by cardiorespiratory (aerobic) endurance, seems to contribute to the development of obesity, type 2 diabetes, hypercholesterolaemia, hypertension, the metabolic syndrome, cardiovascular diseases, and all-cause mortality in both adults and children (18–20). Since the habitual levels of physical activity are closely related to cardiorespiratory fitness, submaximal and maximal exercise testing has become frequently-used indirect physical acti-vity-assessment methods. Results of several studies showed that overweight subjects performed more poorly on cardiorespiratory fitness tests than their thinner counterparts, with low to moderately-high inverse correlations found between cardiorespiratory fitness and adiposity (10, 21, 22).

Although it would appear plausible to assume that the higher level of physical fitness in children results in a more favourable body-composition, data that permit examination of relationship between aerobic fitness and body-fatness in youths are limited and considered controversial (23, 24). Moreover, little is known about the relationship between physical fitness and different body-fatness indicators besides body mass index (BMI), such as waist-circumference or percentage of body-fat, in children. To the best of our knowledge, no data are available examining interdependence between obesity and physical activi-ty indicators in Serbian school children. Therefore, the study was carried out to (a) investigate the prevalence of overweight and obesity among Serbian school children and (b) determine the relationship between physical activity and body-fatness indicators in Serbian school children aged 6-14 years.

## MATERIALS AND METHODS

### Study subjects

The evaluation was performed during September 2007–May 2008 among 1,121 healthy school children of the elementary school programme. Stratified (geographically) random sampling was used in this cross-sectional study. Ten schools were identified from the Department of Education and were randomly selected from the Belgrade borough area of Zvezdara on a proportional basis. Principals of the schools were contacted with the aims of the study explained to both physical education teachers and school administrators. Once a school had agreed to participate in the study, letters to parents were distributed. They completed a sports participation and medical history questionnaire and were informed that they could withdraw from the study at any time, even after giving their written consent. Children with conditions that might have led to limitations in physical activity and mobility were excluded. All the participants were in good health, free from musculoskeletal dysfunctions, and metabolic and heart diseases. None of the subjects was on the medication at the time of the study.

### Experiment design

Each child underwent a one-day testing session. During this session, anthropometric assessment and physical fitness test were carried out. Height was measured using a stadiometer (Seca 202, USA) to the nearest 0.1 cm while body mass was obtained to the nearest 0.1 kg using a calibrated balance beam scale (Avery Ltd, Model 3306 ABV, UK). The subjects were measured with underwear only, in the same state of hydration and nourishment after voiding. All anthropometric measurements were taken between 9 and 11 am after an overnight fasting between 10 and 12 hours. BMI was calculated as weight (kg)/height (m)^2^. Children were considered overweight or obese based on age-specific BMI reference guidelines (Table 1) (25, 26). Waist-circumference was measured using a Gulick anthropometric tape (Creative Health Products, Plymouth, USA) at the level of the narrowest point between the lower costal border and the iliac crest. Skinfold thickness at six sites was obtained using a Harpenden caliper (British Indicators Ltd., St. Albans, UK). The skinfold sites were biceps, triceps, subscapular, suprailiac, abdominal, and medium calf. The landmarks were identified and measured according to Wilmore and Behnke, with the median of three measurements used for representing skinfold thickness (27). The percentage of body-fat was determined according to age- and gender-specific equations (girls, percentage of body-fat=0.610·(triceps skinfold [mm]+calf skinfold [mm])+5.1; boys, percentage of body-fat=0.735·(triceps skinfold [mm]+calf skinfold [mm])+1.0) (28). The same trained technician performed tests on each subject for anthropometric measurements according to the International Society for the Advancement of Kinathropometry. Aerobic fitness was determined using maximal multistage 20-m shuttle-run test (29). Subjects were required to run back and forth on a 20-m course and be on the 20-m line at the same time a beep is emitted from a tape. The frequency of sound-signals increased in such a way that running speed starts at 8.5 km/h and was increased by 0.5 km/h each minute. When the subjects could no longer follow the pace, the stage the subjects were able to run for was recorded and used for calculating the maximal oxygen (VO_2_max) uptake. This test has shown to be valid and reliable for the prediction of the VO_2_max in children (30).

**Table 1.** Reference guidelines for detection of overweight and obesity according to body mass index for-age status categories in children

Status category	Percentile range
Underweight	Less than the 5th percentile
Normal weight	5th to less than the 85th percentile
Overweight	85th to less than the 95th percentile
Obese	Equal to or greater than the 95th percentile

### Statistical analysis

Descriptive statistics were run on all the variables. Statistical significance between continuous variables was assessed using unpaired Student's *t*-test, with p values of less than 0.05 considered statistically significant. Categorical data were evaluated using chi-square (χ^2^) analysis. The relationship between body-fat, BMI, and waist-circumference and VO_2_max was examined using Pearson's product-moment correlation coefficient. Data were analyzed using the SPSS*,* PC program (version 14.0) (SPSS Inc., USA).

### Ethics

All the participants and parents were fully informed verbally and in writing about the nature and demands of the study. All the subjects and parents gave their informed consent and volunteered to participate in the study with the approval of the University's Ethical Advisory Commission in accordance with the Helsinki Declaration.

## RESULTS

**Fig. 1.**
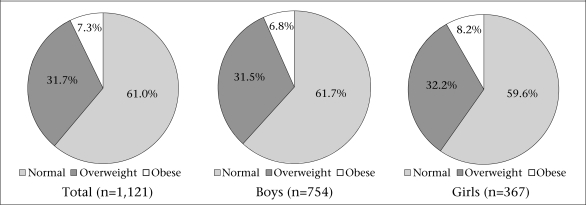
Percentage of overweight and obese subjects

**Fig. 2.**
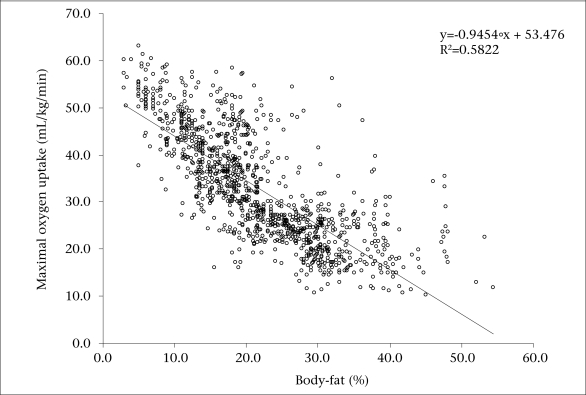
Relationship between body-fat and maximal oxygen uptake (n=1,121)

The prevalence of overweight was 32% and did not vary among the girls and boys (32.2% and 31.5% respectively) (Fig. 1). Significant differences were observed between male and female children regarding the prevalence of obesity (6.8% vs 8.2%, p<0.05, boys and girls respectively). The mean values for anthropometric and physiological data are shown in Table 2. Both boys and girls had comparable age and height. Boys showed significantly lower body-mass, BMI, waist-circumference, sum of six skinfolds, and body-fat compared to their female counterparts (p<0.05). Moreover, boys attained better performance in multistage fitness test and had, therefore, higher VO_2_max (p<0.05). The highest level of weight, BMI, body-fat, and waist-circumference observed in a 14-year old girl (96.3 kg, 40.5 kg/m^2^, 54.5%, and 91.4 cm respectively) implies the existence of extreme obesity in Serbian school children. The relationship between body-fat and VO_2_max is illustrated in Figure 2. The correlation coefficient was moderately high (r=-0.76; p<0.05). There was significant inverse correlation between waist-circumference and VO_2_max (r=-0.43; p<0.05), with insignificant correlation between BMI and VO_2_max (r=-0.09; p*>*0.05).

**Table 2.** Characteristics (mean±SD) of the study subjects by gender

Variable	Boys (n=754)	Girls (n=367)	Range	Total (n=1, 121)
Age (years)	10.4±3.1	10.8±2.8	6.2-14.2	10.5±3.0
Height (cm)	141.6±12.5	142.4±13.7	122.5-188.2	141.9±12.9
Body mass (kg)	37.1±10.8	38.3±11.5*	20.1-96.3	37.5±11.0
BMI (kg/m2)	18.5±2.4	18.9±2.5*	14.3-40.5	18.6±2.4
Waist-circumference (cm)	62.5±8.4	66.1±11.4*	44.5-91.4	64.9±10.4
Sum of 6 skinfolds (mm)	63.2±15.3	74.1±22.6*	18-114	66.8±17.7
Body-fat (%)	20.3±9.1	24.9±9.7*	3.0-54.5	21.8±9.3
VO_2_max (mL/kg/min)	34.1±12.4	30.4±9.6*	10.3-63.2	32.9±11.5

*p<0.05 boys vs girls; BMI:Body mass index; SD=Standard deviation; VO_2_max=Maximal oxygen uptake

## DISCUSSION

The data of the present study clearly demonstrated that a strong negative correlation existed between aerobic fitness and body-fat in Serbian school children aged 6-14 years. The data also demonstrated a high prevalence of both overweight and obese children (39%); the prevalence of overweight was similar among boys and girls. Moreover, girls had inferior cardiorespiratory fitness compared to their male counterparts.

The prevalence of obesity (6.8-8.2%) among the study children did not differ from other national studies in a similar age-group. The prevalence of obesity in children and youths was 8.2% in Aboriginal and non-Aboriginal Canadians (31). Vissers *et al*. reported 7.5% prevalence of obesity in Belgian adolescents, with different prevalence of overweight and obesity among subjects with different types of education (32). The prevalence of obesity among children from Spain was 5-8% (10) while it was 7.9% among boys and 4.7% among girls in Qatari adolescents (33).

Childhood obesity is becoming a global epidemic and is threatening to have reached epidemic proportions in Serbia. When children who were obese and overweight by BMI criteria were considered together, more alarming data generated as higher values of overweight and obese children found (39%) were superior compared to other national studies. About 33% of Baltimore school children were overweight and obese based on the age-specific BMI reference values (34). The prevalence of overweight, including obesity, was higher among boys (25.7%) than among girls (19.1%) in the representative sample of Spanish adolescents (n=2,859) (35). Al-Nakeeb *et al*. indicated that over 37% of boys and girls from Birmingham fell into overweight and obese classifications, with one (22%) in five children having more than 30% body-fat (12). Furthermore, the obese group was almost girl-exclusive (90%). The authors reported that 71% of children had a percentage of body-fat in excess of 20%, which is recognized as the upper end of the optimal range for this age-group. Like previous research, data in the present study indicate that the prevalence of overweight is similar among boys and girls. Girls tend to have a higher prevalence of obesity than boys (8.2% vs 6.8% respectively). Other indices of adiposity, including skinfolds, waist-circumference, and percentage of body-fat, provided rates of preva-lence of overweight and obesity similar to those obtained with age-specific BMI classification. The sex difference in the prevalence of obesity may be related to maturation, growth history, and behavioural and environmental factors, which limits our interpretation and requires more investigation. An interesting gender difference was suggested considering sexual maturation, with early sexual maturation positively associated with overweight and obesity in girls but the associations were reverse for boys (36). In addition, the primary behavioural and environmental factors are food-consumption patterns, sedentary behaviour, socioeconomic status, and micro- (family) and macro- (local society) environment (4). The gradual increase in body-weight that leads to obesity is the consequence of a prolonged positive energy balance, i.e. when energy intake exceeds energy loss. There are many factors that can influence energy balance and, therefore, be identified as contributors to the current obesity epidemic in children, with biological, behavioural, environmental, and social being most cited (37). Although relative contribution of energy intake versus energy loss to the obesity epidemic is a source of continuing debate, available data clearly indicate that physical activity plays an integral role in the prevention of obesity (17, 18, 23). Obese children are at an increased risk of acute medical illnesses and chronic diseases, particularly osteoarthritis, diabetes mellitus, and cardiovascular diseases, which can lead to the poor quality of life, an increased personal and financial burden to individuals, families, and society, and a shortened lifespan (38).

The average VO_2_max for children assessed in the present study (32, 9 mL/kg/min) was lower than previously reported. Studies using procedures for direct measurement of VO_2_max have recorded values between 34 and 58 mL/kg/min for children aged 8-12 years (39–41), with boys having a higher value of VO_2_max than girls. Using the testing protocol similar to the present study, Leger and co-workers reported aerobic capacity from 38 to 52 mL/kg/min for children aged 6-18 years (42). Such discrepancy is likely to be the result of differences in body-composition and level of physical activity, which induce well-established secular trend of decline in aerobic capacity among children (43). The gender differences in VO_2_max found in our study (34.1±12.4 vs 30.4±9.6 for boys and girls respectively) is in accordance with previous studies. Aerobic capacity is consistently greater in boys throughout childhood, with the gender gap widening at the puberty (44). The observed difference has been ascribed to a combination of factors, including body-composition and cardiac size and function (45).

Regular physical activity plays an important role in the maintenance of body-weight and composition and in the regulation of skeletal muscle and adipose tissue metabolism. Several investigators reported an association between physical activity and body-composition in children (5, 10, 12, 35, 37, 46). Precise assessment of habitual physical activity is critical for accurate descriptive epidemiology of the physical activity-obesity relationship for designing appropriate interventions aimed at modifying body-composition and related risk factors and for promoting change in lifestyles (38). However, since physical activity is complex, multidimensional behaviour and precise measurement remain a challenge for practitioners, researchers, and healthcare providers, especially among children.

Self-reported physical activity questionnaire and physiological measures of fitness (direct and indirect assessments of VO_2_max), with several objective techniques, such as heart rate monitors, pedometers, and accelerometers, have been used extensively for the measurement of physical activity in children (47). Although objective techniques are a superior tool for the assessment of physical activity, they are often impractical and time-consuming, thus implying that indirect assessment of physical activity could be more appropriate in studies with a large sample. Moreover, study of aerobic fitness and body-fat is probably a more valid approach than measurement of self-reported physical activity and body-weight, particularly in children (48, 49).

It has been reported that overweight and obese children showed a lower physical fitness (cardiores-piratory endurance) than normal children (50, 51). However, studies analyzing an association between cardiorespiratory fitness and different measures of body-composition in this population are scarce. Our results showed a strong inverse relationship (r=-0.76) between aerobic fitness and percentage of body-fat in both boys and girls. The results in terms of other anthropometrical variables showed both significant (with waist-circumference) and insignificant (with BMI) relationship with cardiorespiratory fitness, which has also been reported earlier in children and adolescents (46, 48). Klasson-Heggebo *et al*. found a curvilinear-graded relationship between cardiorespiratory fitness and waist-circumference and sum of skinfolds in 4,072 children and adolescents aged 9 and 15 years respectively, from Denmark, Portugal, Estonia, and Norway, with abundant data showing a significant inverse relationship between physical activity level and body-fat percentage (50). Hence, it is not surprising that people who have sedentary lifestyles also have low levels of fitness and excessive body-fat (48). Given that this study was cross-sectional in design, cause-and-effect conclusions are not warranted. However, it appears that the goal of favourably altering adiposity in children should begin with increasing physical activity and fitness, which, in turn, will lead to reductions in body-fat. Moreover, children who improve their cardiorespiratory fitness during childhood have less overall adiposity and less abdominal adiposity than their counterparts during adolescence and adulthood (3). Participation in vigorous physical activities has been shown to relate inversely to fat deposition in both children and adults (5, 18, 38, 52). Due to the fact that BMI poorly correlates with aerobic fitness, other indicators of adiposity (body-fat, waist-circumference) rather than weight has been shown to be associated with cardiorespiratory fitness (19, 37). Several investigators provided evidence that a combination of simple measures, such as triceps and calf skinfolds, waist-circumference, and perhaps fitness, should be used in clinical settings to identify children with high risk of obesity (49, 51, 53).

Our results suggest that cardiorespiratory fitness as an indicator of physical activity is linked to the increased level of adiposity in children. Inactivi-ty is only one of the factors interconnected with obesity; however, it is perhaps one of the easiest to modify (37). Considering that physical education is the only source of regular physical activity for many children (54), improvement in physical education class may be prudent. The main aim of such classes should be to engage children in health-providing physical activity levels in contrast to the present curriculum design for movement skills development. Moreover, it has been proved that physical education can have an important role in promoting participation of children in extra-curricular health-enhancing physical activity (55, 56). Guidelines for physical activity in youths recommend involvement in moderate to vigorous physical activities for at least 60 minutes a day for health promotion and from a weight-control perspective (18). According to the results of the present study and the fact that Serbian academic curriculum includes only 90-minute physical activity per week, initiatives should be put forward to promote physical activity in Serbian children in both school and out-of-school environment.

### Limitations

A clear understanding of the relationship between physical activity and/or physical fitness and body-composition in children in the present study may not be possible due to several limitations. Despite the value of endurance test for indirect measurement of physical activity in children, it has been stated that more accurate measures of physical activity are required to determine if an association truly exists between activity level of children and their aerobic fitness on obesity (52). Moreover, the analysis of fitness-fatness association will be further improved with adjustment for pubertal development factors, which requires further investigation. The need for parental agreement and consent might have also lead to bias, with those parents more committed to physical activity agreeing to participation of their children (51). Finally, it can be said that BMI is a relatively weak substitute for measuring the percentage of body-fat and, thereby, obesity (37). Combination of BMI with body-fat and physical fitness needs to be used for establishing a more relevant standard definition for childhood overweight and obesity worldwide (53).

### Conclusions

The results of the present study showed that body-fat and waist-circumference negatively correlated with aerobic fitness, suggesting that children who have high cardiorespiratory fitness during childhood have less overall adiposity and less abdominal adiposity than their unfit counterparts. Furthermore, the prevalence of overweight and obesity in Serbian school children was high, with girls having higher obesity rates than boys. Although there are many factors that can contribute to obesity among children, this study emphasizes the necessity to develop interventions to improve physical fitness in children and to prevent the increase of childhood obesity.

## ACKNOWLEDGEMENTS

The Serbian Ministry of Science supported the study (Grant No. 145082). The authors declare no conflict of interest.
